# Study on the Stability of Bio-Oil Modified Prime Coat Oil Based on Molecular Dynamics

**DOI:** 10.3390/ma15196737

**Published:** 2022-09-28

**Authors:** Shuang Shi, Lanqin Lin, Zhaoguang Hu, Linhao Gu, Yanning Zhang

**Affiliations:** 1 School of Transportation, Southeast University, Southeast University Road #2, Nanjing 211189, China; 2 China Road and Bridge Corporation, 88 Outer Andingmen Street, Beijing 100011, China

**Keywords:** road engineering, stability, molecular dynamic, bio-oil asphalt/water emulsion, particle size, infrared spectrum analysis

## Abstract

To explore the effect of different emulsifier contents on the stability performance of biomass-emulsified asphalt, three types of emulsified asphalt with 1%, 3%, and 5% anionic emulsifiers were prepared and analyzed by molecular dynamics simulation and macroscopic experiments. Firstly, we used molecular simulation software (Material Studio, MS) to construct a model of biomass-emulsified asphalt with different emulsifier contents and analyzed the microscopic mechanism of the emulsifier to improve the stability of the emulsified asphalt by the radial distribution function, interaction energy, interfacial layer thickness, and solubility parameters of the emulsified asphalt system with different emulsifier contents. The results were validated by macro and micro tests including storage stability, particle size determination, and infrared spectroscopy. The results show that at low emulsifier contents, the emulsifier can reduce the interfacial tension between the oil–water interface and expand the transition region between the two phases (interfacial layer thickness), which will prevent interparticle agglomeration and reduce the emulsion particle size, thus reducing the settling rate and ensuring the stability of the emulsion. When the emulsifier content is further increased beyond the critical micelle concentration, the emulsifiers will agglomerate with each other and show larger peaks in the radial distribution function, and the phenomenon of emulsifier agglomeration will appear in the five-day storage stability test, resulting in a corresponding decrease in the proximity of the infrared absorption peak area ratio in the same wavelength band of the upper and lower layers of the biomass-emulsified asphalt, and the emulsion stability decreases instead.

## 1. Introduction

As the country’s economy continues to develop, the convenience and comfort of transportation have become key factors in determining the further improvement of the country’s economy. In recent years, China’s transportation system has also become more and more developed, road density is increasing, and the construction of highways is becoming more and more advanced. However, the material properties between the two structural layers of roads have large differences, and the layers are not in a completely continuous state; when subjected to a large horizontal shear stress, the surface layer will occur nudging, cracking, and other pavement deteriorations. Therefore, the current highway construction process often uses prime coat oil construction for interlayer disposal to achieve the following functions: First, the consolidation of loose aggregates on the surface of the grass-roots level improves the surface strength of the base layer level. Second, sealing the grass-roots level and playing a role in waterproofing. Third, increasing the adhesion between the base layer level and the surface layer ensures continuity between the base layer, so that the neutral surface is always located in the semi-rigid base layer, allowing the asphalt surface layer to withstand the larger layer bottom bending, tensile stress, and fatigue damage [1,2,3,4].

However, the prime coat oil needs to have a certain storage stability and sufficient penetration depth to ensure the use of the construction process and the pavement structure to play the above role. An asphalt/water emulsion is more stable after breaking the emulsion, will not volatilize, and has high safety and good economic and environmental benefits; furthermore, with the continuous optimization of the production process of asphalt/water emulsion and continued improvement of the production process, the application of an asphalt/water emulsion in the road structure has become more widespread, so in the prime coat oil, an asphalt/water emulsion has also begun to replace kerosene-diluted asphalt to optimize road performance [5,6].

In addition, with the implementation of the “double carbon” plan advocated by the state, the recycling of resources and the environmental protection of resources have put forward higher requirements, and the extraction of biomass materials from animal and plant remains, restaurant waste oil, used handicrafts, and other resources has gradually attracted the attention of the road engineering industry [7]. Researchers at home and abroad have modified asphalt with biomass materials, and the feasibility, self-healing, anti-aging, and anti-rutting properties of biomass asphalt have been fully investigated from multiple perspectives, and it has been found that biomass materials can indeed enhance some specific properties of asphalt materials [8,9,10,11,12]. Through preliminary research and experimental studies, it was found that adding biomass oil as a penetrating agent to asphalt/water emulsions can also effectively enhance the penetration depth of prime coat oils, but there is a lack of corresponding micro-level studies around how much emulsifier dosage can make the biomass prime coat oil achieve better stability overall. If the stability of biomass prime coat oils can be investigated from a microscopic point of view with different emulsifier contents, a positive impact on the optimization of prime coat oils and their application in asphalt pavements will be achieved. For microscopic-level research, molecular dynamics has simulation characteristics at the nanoscale, which can simulate the physical motion of system atoms or molecules and then effectively calculate the intermolecular interactions and system energy, and the method can track the dynamic evolution process of complex systems over time and reveal the adsorption properties and action mechanism of molecules at interfaces in minute time scales [13]. Fan et al. [14] used molecular simulation software to construct a water/emulsifier/asphalt system for molecular dynamics simulation and studied the effect of the interaction between asphalt and emulsifier on asphalt–water interface properties by parameters such as interface formation energy, interfacial layer thickness, and diffusion coefficient. He et al. [15] conducted a comprehensive analysis of the molecular dynamics of an asphalt system, constructed the general equilibrium steps of the molecular dynamics model of asphalt systems, proposing to verify the validity of the model with density, glass transition temperature, viscosity, etc., and summarized the progress of the molecular dynamics approach to study the behavior mechanism of asphalt nano aggregation, self-healing, and modification. Zhang et al. [16] analyzed the “self-healing” mechanism of graphene-microencapsulated asphalt by infrared spectroscopy, fluorescence microscopy, and molecular dynamics simulation. Yu et al. [17] added bio-based epoxy soybean oil to cold mix epoxy asphalt and found that the difference between the cohesive energy density and solubility of asphalt and epoxy monomer decreased, and the interaction energy of the whole system increased by Fourier variation infrared spectroscopy and molecular dynamics simulation. Kapyelata et al. [18] utilized the simulation technique to study the carbon nanotube/recycled polyethylene asphalt interaction and found that carbon nanotubes can promote the distribution of asphaltene fractions, thus improving the storage stability of the recycled polyethylene-modified asphalt. Based on the above study, this paper will investigate the effect of emulsifier content on the stability of a biomass asphalt/water emulsion based on the molecular dynamic (MD) simulation technique combined with macro and micro tests. At present, most studies on the stability of asphalt/water emulsions based on molecular dynamics focus on how the emulsifier reduces the interfacial tension between the asphalt and water phases, and the commonly used characterization parameters are interaction energy, interfacial layer thickness, and compatibility parameters. In this paper, the effect of emulsifier dosage on the stability of a biomass anionic asphalt/water emulsion will be investigated from three perspectives, namely, interaction energy, interfacial layer thickness, and radial distribution function, using MS, and the optimal emulsifier dosage will be determined and validated by macro and microscopic tests of storage stability, particle size analysis, and IR spectroscopy.

## 2. Test Material and Microscopic Model

### 2.1. Test Materials and Instruments

#### 2.1.1. Instruments and Sample Preparation

In this paper, three kinds of macro or micro tests were involved, namely, infrared spectroscopy, laser particle size measurement, and storage stability experiments. For the accuracy of the test results, three parallel tests were conducted for each test, and the average of the three test results was taken as the final result.

For the infrared tests, we used a Nicolet iS10 Fourier infrared spectrometer, and the minimum resolution was 0.5 cm^−1^, the experimental parameters were set as 64 scans and 4 cm^−1^ resolutions, and then omic software was used to perform baseline correction and smoothing on the obtained IR spectral curves. Finally, the inflection point tangent was used to measure the infrared characteristic peak area of the pre-processed IR spectral map.

For particle size data acquisition, an LS200 laser particle sizer was used in this paper. The instrument has an analysis range of 0.1 μm to 750 μm and can derive parameters such as refractive index as well as shading ratio for various media. The test parameters were set as follows: the background sampling time was 10 s, the single sampling time was also set to 10 s, and the result type was volume (V).

Storage stability experiments were performed by injecting 250 mL of emulsified bitumen solution into stability test tubes and leaving them on a smooth platform for 5 days. After five days, the emulsified bitumen solution was exported through the upper and lower branch tubes and evaporated. Finally, the storage stability of the asphalt/water emulsion was determined through the upper and lower branches of the asphalt/water emulsion solid content difference. 

#### 2.1.2. Materials

In this study, Korea SK-70# asphalt was chosen as the base asphalt; its physical properties are shown in Table 1. All indicators met the relevant technical regulations in China.

In addition, this paper used self-developed anionic emulsifier to prepare an asphalt/water emulsion, the main component was which was sodium dodecyl diphenyl ether desulphonated. According to the existing basic test results, 3% of the emulsifier content of the asphalt/water emulsion samples prepared more uniform particles and the emulsification effect was better, so in the test group, 1%, 3%, and 5% were set as the emulsification content.

Biomass oil was produced independently, and its main components were soft fatty acid, linolenic acid, oleic acid, and stearic acid, and the specific gravity ratio of the components was 14:33:37:15. The best dosing was studied through indoor tests and was determined as asphalt:biomass oil = 10:1.

### 2.2. Molecular Model of Biomass Asphalt/Water Emulsion

#### 2.2.1. Determination of Asphalt/Water Emulsion Composition and Model Proportioning Calculation

In this paper, the average molecular structure model of asphalt consisting of twelve components proposed by Li et al. based on SARA four-component analysis was chosen as the molecular model of matrix asphalt [19,20,21], and the ratio of the twelve components was adjusted according to the four-component ratio of SK-70# matrix asphalt [22] (Table 2), and the final molecular composition of each component’s molecular composition is shown in Table 3. Then the emulsifier molecules, water molecules, and biomass oil molecules were assigned according to 50% solids content and 10:1 asphalt/biomass oil, and the asphalt/water emulsions with 1%, 3%, and 5% emulsifier content were established, respectively, with the specific molecular parameters shown in Table 4.

#### 2.2.2. Molecular Modeling of Asphalt/Water Emulsion

**Figure 1:**
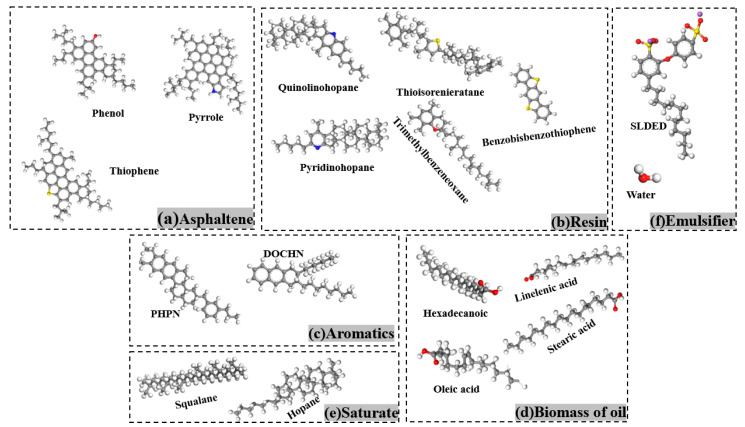
Composition diagram of bio-oil asphalt/water emulsion model.

In this study, the kinetic calculation of molecular systems was carried out on the Material studio 2021 computational simulation platform, and the COMPASS II force field environment was selected, which can classify organic and inorganic molecular systems. Firstly, the molecular models were constructed under the Visualizer module of Material studio, according to Figure 1, and the bond angles and bond lengths were adjusted using the Clean function. Then, the Amorphous Cell module was used to encapsulate the molecular models by the number of ratios in Table 3 and Table 4, and finally the Geometry Optimize function in the Forcite module was used to optimize each box to achieve the most stable configuration (lowest molecular energy).

In addition, due to the relatively complex configuration of biomass asphalt, it was necessary to anneal it to eliminate the unreasonable internal structure, and finally to perform two separate kinetic calculations of different systems to make it closer to the actual material properties and ensure the accuracy of the simulation. To make sure that the emulsifier could better achieve the effect of reducing the interfacial tension between water and biomass asphalt, the build layer’s function was used to assemble the three components in the order of water, emulsifier, and biomass asphalt, and in the last, a vacuum layer of 30 Å was reserved to prevent the effect of periodic boundary conditions.

## 3. Emulsifier Dosing on the Stability of the Biomass Asphalt/Water Emulsion

### 3.1. Radial Distribution Function

**Figure 2:**
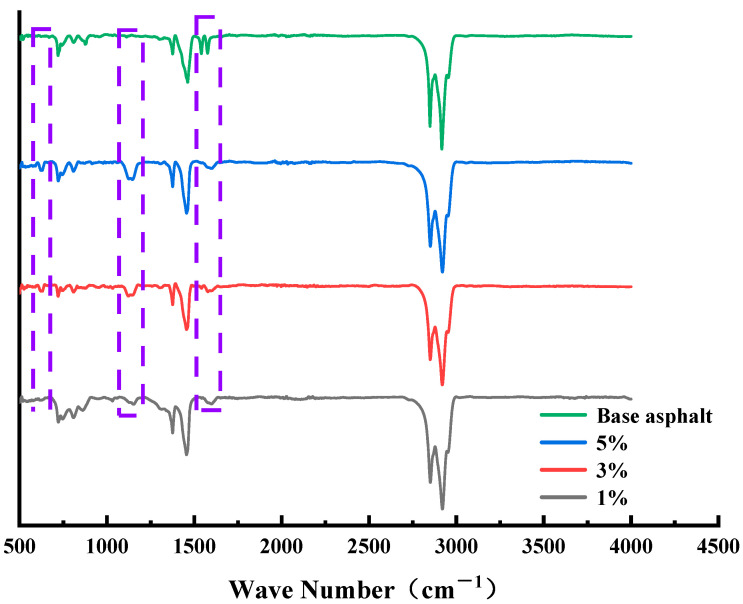
Infrared spectra of biomass asphalt/water emulsion with different emulsifier contents (The purple dashed line represents the main characteristic peak changes occurring in the emulsified asphalt relative to the matrix asphalt).

Before conducting the molecular dynamics simulations, this paper first analyzed the effect of emulsifiers on matrix asphalt roughly by infrared spectroscopy; the test results are shown in Figure 2.

The common characteristic peaks of each control group in the figure were attributed as follows: the anti-symmetric stretching vibration of methylene (–CH_2_–) near wave number 2948 cm^−1^ and the symmetric stretching vibration of –CH_2_– near wave number 2849 cm^−1^, indicating that the alkyl chains in the alkane compounds in the control group were arranged in an orderly manner at this time. The alkane methylene (–CH_3_) asymmetric variable angle vibration was near wave number 1455 cm^−1^, the alkane methylene symmetric variable angle vibration peak was near wave number 1375 cm^−1^, and wave numbers 803 cm^−1^, 745 cm^−1^, and 727 cm^−1^ were the range of wave numbers in the fingerprint region, which is the benzene ring substitution region, and the characteristic peaks appearing in this range were caused by the C–H bond on the benzene ring swinging out of plane vibrations. In addition to the same characteristic peaks mentioned above, it was seen that the matrix asphalt had a double-peak structure at wave numbers 1536 cm^−1^ and 1574 cm^−1^ for the stretching vibration of the double bond and the bending vibration of H–O, H–N. However, with the addition of the emulsifier and the increase of dosage, it was seen that the double peaks gradually evolved into single peaks and move towards the vicinity of wave number 1593 cm^−1^. The reason for this situation may be caused by the induction of the C=C double bond of the benzene ring in the asphalt with the O atom of the emulsifier molecule, resulting in the high spectral band of the splitting to about 1593 cm^−1^. The evaporated residues of the biomass asphalt/water emulsion around wave numbers 1151 cm^−1^ and 1124 cm^−1^ showed wave peaks at these two locations, and the peak intensity became progressively larger with increasing emulsifier content as a result of SO3 symmetric stretching vibrations of aromatic sulfonates in more emulsifiers. Furthermore, near the wave number 628 cm^−1^, with the increase of emulsifier dosage, there was a wave peak with a gradual increase of signal intensity, which is part of the C–S stretching vibration. Simulation results also proved that 3% emulsifier content was near the critical micelle concentration of the anionic emulsifier used in this paper, which had good stability performance and could guide the amount of emulsifier.

**Figure 3:**
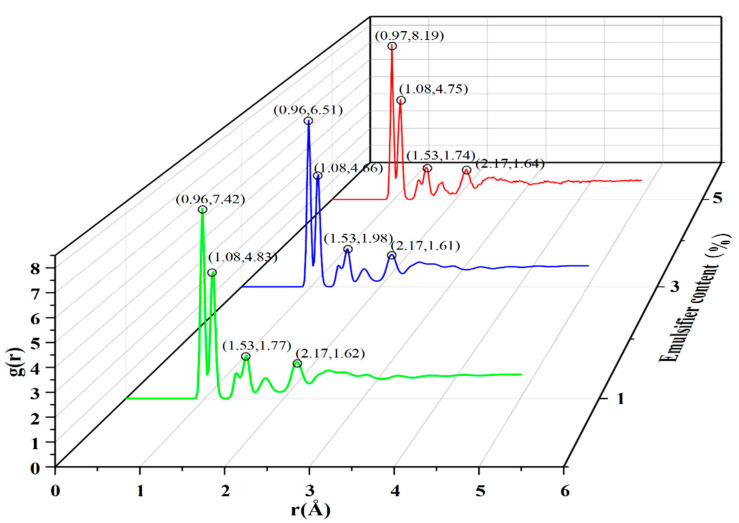
Radial distribution function of asphalt/water emulsion with different emulsifier contents.

This paper first used the molecular simulation method to analyze the radial distribution function of the biomass asphalt/water emulsion; the radial distribution function characterizes the probability of occurrence of other particles around a particle and thus can be used to reflect the stacking of the polymer model. In this paper, the radial distribution function was calculated for three emulsifier contents of a biomass asphalt/water emulsion, and the results are shown in Figure 3.

**Figure 4:**
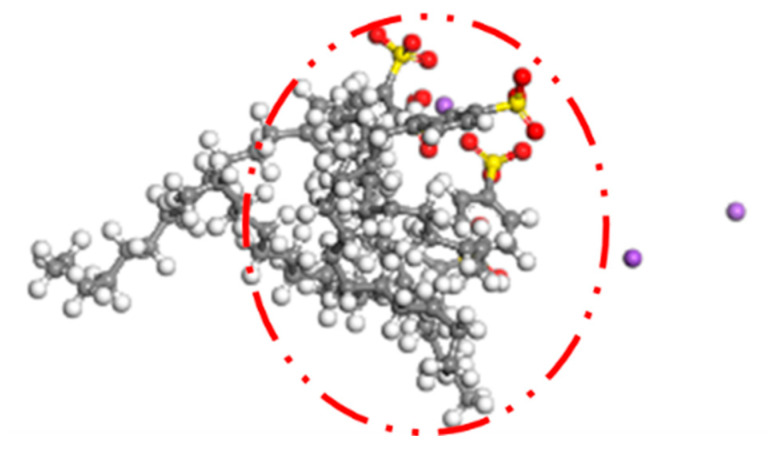
Agglomeration of emulsifier with 5% emulsifier content.

As seen in Figure 3, the overall trend of the radial distribution function corresponding to the three emulsifier contents was the same, all forming four more obvious peaks, with the first and second peaks being significantly larger than the peaks of the other two peaks. The first peak position of these contents of emulsifier was located at 0.96 Å, and the peak size showed that 1% > 5% > 3%, while for the other peaks, respectively, they appeared at 1.08 Å, 1.53 Å, and 2.17 Å, and the values were basically similar. From the results of the radial distribution function, it can be seen that the agglomeration phenomenon of biomass asphalt/water emulsion appeared to be weakened and then enhanced during the increase of emulsifier addition, which is because the emulsifier has hydrophilic chain segments at one end and hydrophobic chain segments at the other end with good surface activity, and its adsorption on the two-phase interfacial film is enhanced with the increase of emulsifier dosage, and the emulsifier spontaneously generates a water interface between the two phases, which makes the surface-free energy function of the emulsion as low as possible to give the emulsion high stability. However, as the emulsifier concentration increases to a certain value, the emulsifier molecules start to cluster to form aggregates, with the lipophilic chain segments clustering together and the hydrophilic chain segments reaching outward to the aqueous phase to form the so-called micelles, and the lowest emulsifier concentration that can form micelles is the critical micelle concentration, referred to as the CMC value [23,24,25]. As shown in Figure 4, the biomass asphalt and water molecules of the biomass asphalt/water emulsion after molecular dynamics simulations were hidden, and through observation of the distribution and structure of the emulsifier, it found that with the increase of the emulsifier dosage, at 5% emulsifier dosage, after kinetic calculations, a part of the emulsifier molecules started to approach each other between the lipophilic groups, and the emulsifier molecules started to cluster. This conclusion is similar to Quan Xiujie’s radial distribution function simulation results for emulsified asphalt with different emulsifiers. The peak value of emulsified asphalt in the first stage will increase first and then decrease with the increase of emulsifier content [26].

### 3.2. Interaction Energy

During molecular dynamics simulations, the intermolecular interaction energy is often used to quantitatively characterize the strength of intermolecular interactions and thus predict the compatibility between materials of the blended system. In this paper, the total intermolecular interaction energy, van der Waals interaction energy, and electrostatic interaction energy were used as evaluation indicators to evaluate the stability of the system [27,28,29,30,31].

**Figure 5:**
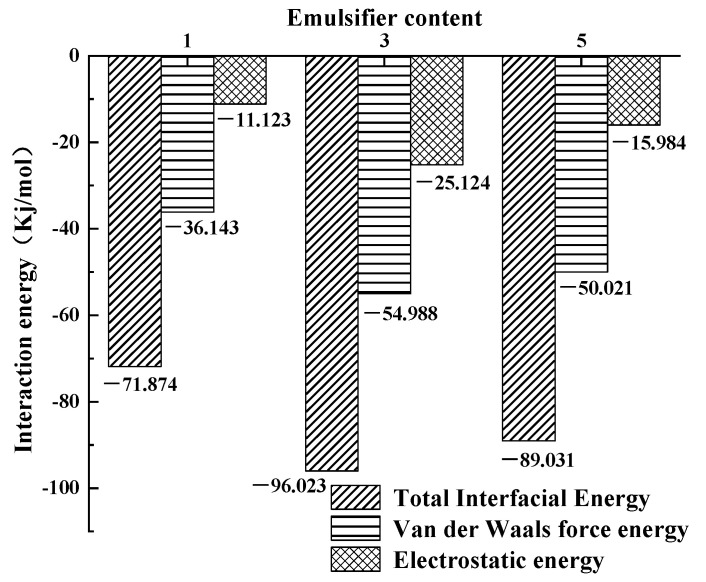
Interaction energy of asphalt/water emulsion with different emulsifier contents.

The energy values of the biomass asphalt/water phase, biomass asphalt, and the water phase after kinetic simulation were each calculated by the Energy function in the Forcite module and calculated according to the interaction energy calculation equation; the results are shown in Figure 5.

It can be clearly seen that the total interfacial action energy, van der Waals force action energy, and electrostatic force action energy of the biomass asphalt/water emulsion at the three emulsifier contents were all negative, indicating that the emulsifier played a role in reducing the energy at the interface between the biomass asphalt phase and the water phase at all three emulsifier dosages, thus reducing the interfacial tension between them. However, under the same simulation conditions, different emulsifier contents showed different action energies, again indicating that the emulsifier content had some influence on the stability at the interface. With the increase of the emulsifier content, all three action energies showed a trend of first increasing and then decreasing, i.e., the stability of biomass asphalt/water emulsion would become stable at the beginning due to the reduction of interfacial tension by the emulsifier, but when the amount of emulsifier further increased beyond the so-called critical micelle concentration, the whole system would become unstable. This is because the force between the hydrophilic group polar head of the emulsifier molecule and the water molecule was mainly van der Waals force, while the anionic emulsifier molecule itself had a negative charge, resulting in a strong electrostatic force of mutual attraction between it and the positively charged asphalt molecule, thus having the ability to strengthen the interaction between the oil and water phases. However, as mentioned above, when the emulsifier content was too high, the emulsifier flocculation phenomenon occurred, and then this part of the emulsifier could not play or completely play its role, the interaction between the oil and water phase weakening instead.

**Figure 6:**
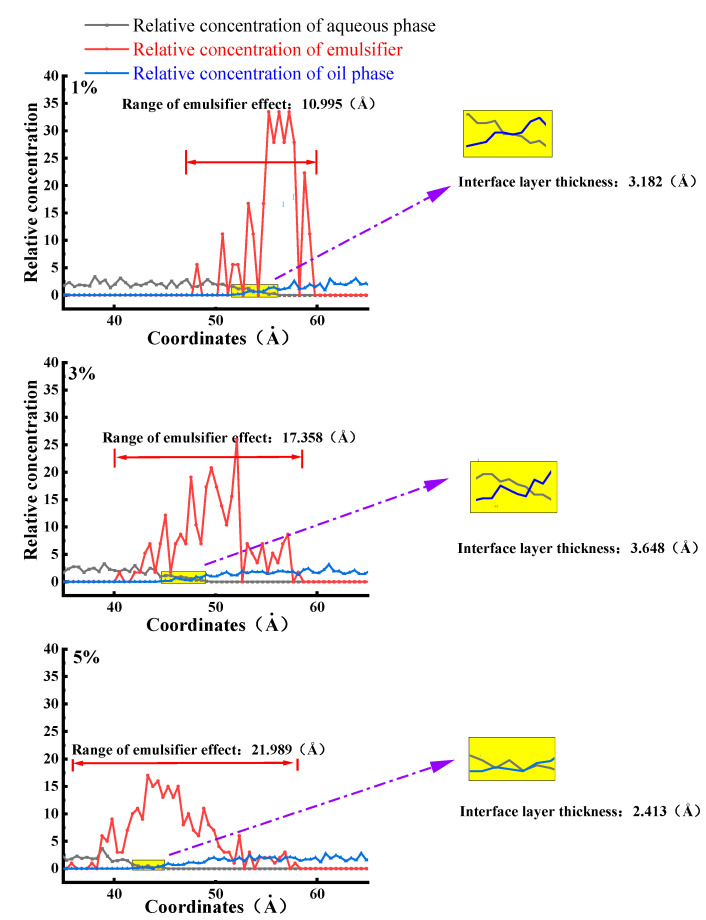
Concentration distribution of each component of bio-oil asphalt/water emulsion along the *z*-axis.

In addition, as the emulsifier strengthened the interaction between the oil and water phases, the oil and water phases penetrated each other, the thickness and volume of the interface layer formed at the interface increased, the activation of the emulsifier on the interface became stronger, and the stability of the asphalt/water emulsion was better [27]. Therefore, this paper further analyzed the stability situation with the help of molecular dynamics simulations to calculate the relative concentration distribution curves of biomass asphalt, water, and emulsifier to characterize the interfacial layer thickness. To quantitatively describe the effect of different emulsifier contents on the oil/water interfacial layer, the thickness of the oil/water interfacial layer was defined using the “10–90%” thickness principle in this paper [30]. The thickness range of the interfacial layer is roughly marked with yellow squares in Figure 6, and the black arrows point to the enlarged view of the interfacial layer, and in addition, the simulation and calculation results are shown in Figure 6. According to the change of temperature and emulsifier content, Fan Weiyu [14] also used molecular dynamics simulation to launch a simulation containing emulsified asphalt’s asphalt/water interfacial energy and diffusion coefficient in the corresponding research. Finally, he also drew the conclusion, which is the same as the one in this paper, that the emulsifier can abate the repulsion between the asphalt/water interface, but this effect will increase with the increase of emulsifier content and then begin a weakening trend, that is, there is too much emulsifier, but it cannot make asphalt and water increase their mutual solubilization, which means a poor emulsifying effect.

**Figure 7:**
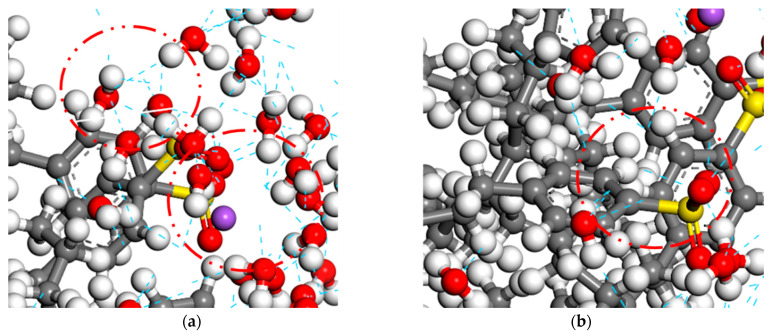
Hydrogen bonds between molecules of bio-oil asphalt/water emulsion. (**a**) Hydrogen bond between water and emulsifier. (**b**) Hydrogen bond between bio-oil asphalt and emulsifier.

As seen from the figure, water molecules and biomass asphalt molecules in the simulation both moved to the middle of the system, and the distribution area of the water phase and the oil phase appeared to have a certain degree of overlap, representing a certain degree of mutual solubility between the water phase and the oil phase. The emulsifier monolayer film was distributed in the transition region of the asphalt–water interface, because the hydrophilic group of the emulsifier had a strong van der Waals force effect with the water molecules, causing some water molecules to be adsorbed in the surrounding polar base, thus expanding the transition region of the water molecule interface. Furthermore, the emulsifier had a strong negative charge, which extended to the oil phase, and the two had a strong electrostatic adsorption effect, so that oil molecules and the lipophilic base also had a certain amount of mutual solubility, thus increasing the transition region of the oil phase, and furthermore, there was a certain amount of mutual solubility between oil and water, causing the tension of the asphalt–water interface to be reduced. Therefore, the wider the thickness of the interfacial layer, the larger the transition area between the water phase and the oil phase, the weaker the interfacial repulsion between oil and water, and the lower the interfacial tension between oil and water. The thickness of the interfacial layer increased at first with the amount of emulsifier from 1% to 3%. However, when the amount was further increased, the thickness of the interfacial layer decreased. This is because when the emulsifier content reached the critical micelle concentration, with continued increases in the emulsifier content, the emulsifier would agglomerate, and due to the stronger hydrogen bonding of water molecules to emulsifier molecules compared to asphalt molecules as Figure 7 shows, the adsorption at the interface would no longer increase, but the emulsifier would be concentrated in the water phase, and thus the interface layer thickness would tend to increase in flatness or due to the strong van der Waals forces between the emulsifier molecules, so that the original adsorption at the interface part of the emulsifier molecules occurred in the water phase, thus reducing the thickness of the interface layer. The present results also demonstrated again that 3% emulsifier content gave the best stability of the biomass asphalt/water emulsion among the three emulsifier dosages.

### 3.3. Particle Size Measurement

According to Stokes’ law, as shown in Equation (1):

(1) $$v=\frac{2gr^{2}(\rho_{1}-\rho_{2})}{9\eta }$$

where *v* is the settling velocity of asphalt/water emulsion particles; *g* is the acceleration of gravity; *r* is the radius of asphalt/water emulsion particles; *ρ*_1_ is the relative density of asphalt; *ρ*_2_ is the relative density of the water phase; and η is the viscosity of the water phase.

Asphalt/water emulsion particles will settle under the action of gravity, and their settling rate is proportional to the square of the particle size, which means that the particle size of an asphalt/water emulsion has the greatest influence on its storage stability, so the stability performance of biomass asphalt/water emulsions can be evaluated by the particle size test. In this paper, the particle sizes of three biomass asphalt/water emulsions were tested by an LS-200 laser particle size meter, and the results are shown in Table 5.

Different opinions have been put forward by domestic and foreign scholars as to which particle size index is a better guide for the performance of asphalt/water emulsions, mainly focusing on D_50_, volume mean particle size, and particle size span (span) [32,33,34,35,36,37], while the definition of particle size span is shown in Equation (2):

(2) $$Span=\frac{D_{90}-D_{10}}{D_{50}}$$

**Figure 8:**
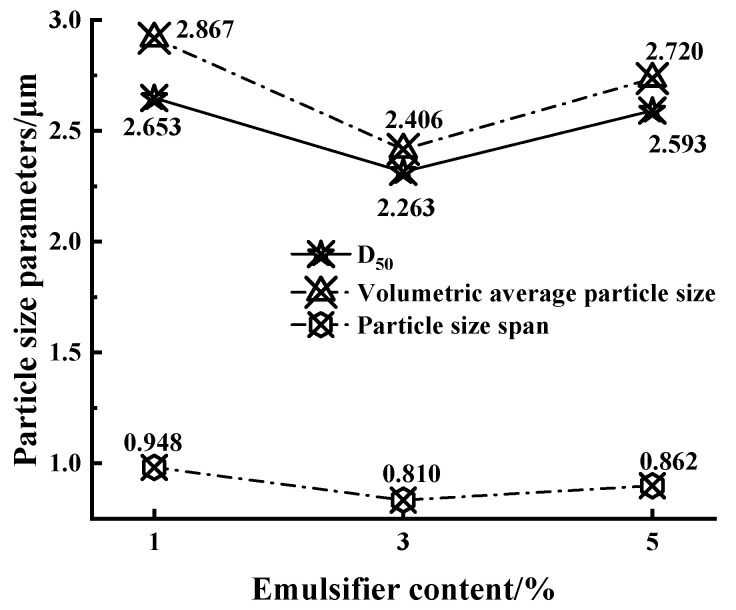
Particle size indexes of bio-oil asphalt/water emulsion with different emulsifier contents.

In this paper, these three parameters were also selected as evaluation indexes for the particle size of the asphalt/water emulsion, and the results are shown in Figure 8.

Based on previous studies, it was mostly believed that the average particle size of asphalt/water emulsion particles should be less than 2–4 μm to achieve better stability performance and permeability [32,33,37]; as shown in Figure 8, the prepared asphalt/water emulsion met the requirements in terms of particle size. However, in contrast, in terms of the D50 and the volume average particle size, it was found that with the increase of the amount of emulsifier, the two particle size indicators showed a trend of firstly decreasing and then increasing, which is consistent with the results of the interaction energy simulation, where the thickness of the interfacial layer, with an increasing content of emulsifier, firstly decreased and then increased, and there could be different degrees of thickness of the interfacial film between oil and water to prevent particle aggregation forming larger asphalt/water emulsion particles. In addition, because the emulsifier content was lower, the ionization of the emulsifier molecules on the surface of the particles with a charge, the electrostatic repulsion of the same charge to prevent asphalt particles, and, within a certain range, to increase the amount of emulsifier, could make the asphalt particles increase the charge strength, resulting in electrostatic repulsion also becoming stronger, and the storage stability of the asphalt/water emulsion becoming better. However, when the amount of emulsifier exceeded the critical micelle concentration, the particle size of the asphalt/water emulsion increased with the amount of emulsifier and became larger, because when the concentration of emulsifier reached the critical micelle concentration, the asphalt–water interfacial tension no longer decreased and tended to stabilize, and the emulsifier in addition to the asphalt surface aggregated into a film, the excess emulsifier molecules formed their own lipophilic base inward, and the hydrophilic base formed outward multi-molecular aggregates, that is, micelles or micelles. The formation of the average particle size of the emulsion increased. The particle size span reflected the uniformity of particle size distribution of the asphalt/water emulsion, and the sample with 3% emulsifier dosage also had the best uniformity at the same time. From the particle size perspective, this indicates that the biomass asphalt/water emulsion with 3% emulsifier dosage had better stability.

### 3.4. Storage Stability

**Figure 9:**
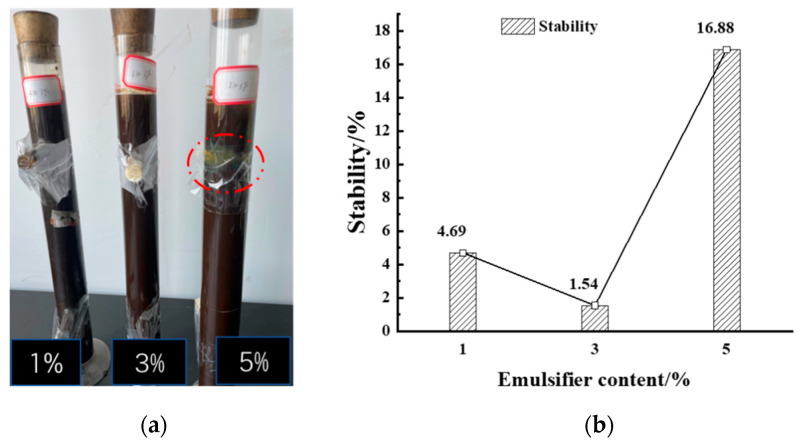
Effect of emulsifier dosing on the storage stability performance of biomass asphalt/water emulsion. (**a**) Flocculation of Asphalt/water emulsion. (**b**) Stability.

The storage stability test mainly consisted of the sample closed stability test tube being placed on the test tube clamp at room temperature for 5 days and nights; a resting process; daily observation of the emulsion to determine whether there was delamination, precipitation, or discoloration, etc.; and finally, analyzing the upper and lower branches of the asphalt/water emulsion solid content difference to determine the storage stability of the asphalt/water emulsion, and the three different emulsifier contents of asphalt/water emulsion storage stability, as shown in Figure 9.

**Figure 10:**
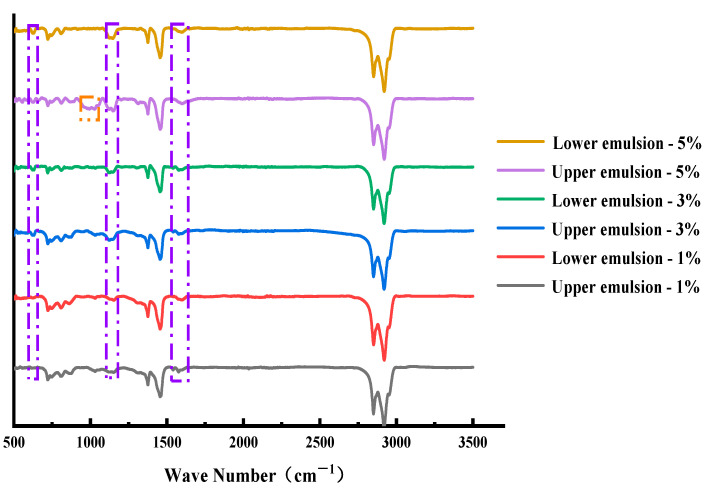
Infrared spectra of bio-oil asphalt/water emulsion at different layers with different emulsifier contents (The purple dashed line represents the main characteristic peak changes occurring in the emulsified asphalt relative to the matrix asphalt).

As can be seen from Figure 9, when the emulsifier content was relatively small, the prepared asphalt/water emulsion had good storage stability and could meet the values required by the specification. With the increase of the amount of emulsifier, the storage stability of the biomass asphalt/water emulsion showed a trend of decreasing and then increasing, which was similar to the emulsifier agglomeration obtained from the simulation results of the radial distribution function, namely, the peak of the first peak of the test group with 5% emulsifier content in the simulation was significantly higher than the other two groups, which was also reflected in the stability experiments, as shown in Figure 9a, At 5% emulsifier content, the emulsifier showed a significant agglomeration into clusters. Therefore, the stability test tube test also proved that a certain degree of increase in the emulsifier content could improve the storage stability of asphalt/water emulsions, but when the emulsifier content was further increased beyond the critical micelle concentration, the asphalt/water emulsion appeared to agglomerate and flocculate, thus affecting the stability and related properties. However, the accuracy of the stability test tube test was affected by the placement and evaporation of the test tube and the lack of analysis of the changes in the composition of the asphalt/water emulsion after modification and the differences in the composition of the upper and lower branches after dissociation. Figure 10 shows the infrared spectra of the evaporated residues of the asphalt/water emulsion in the upper and lower branches after the 5 d stability test tube with different emulsifiers.

The basic wave peaks on the wave spectrum are described in Section 2.1.1 except that the evaporated residue of the upper biomass asphalt/water emulsion with 5% emulsifier content showed significant wave peaks at wave numbers 999 cm^−1^ and 1028 cm^−1^ that other controls did not have, as C–S vibrations between the S atom of the emulsifier and the C atom of the aromatic ring, which corresponded to the storage stability test where the upper 5% asphalt/water emulsion solution showed. The emulsifier agglomeration phenomenon corresponded to the phenomenon that when the emulsifier is mixed too much, the excess emulsifier will be aggregated in the water phase and thus suspended in the upper part of the biomass asphalt/water emulsion, resulting in a large gap between the upper and lower solution components and a decrease in storage stability. On the contrary, when the emulsifier content was 1% and 3%, the IR spectra of the upper and lower solutions did not show much difference, which proved that the prepared asphalt/water emulsion had good storage stability [38].

**Figure 11:**
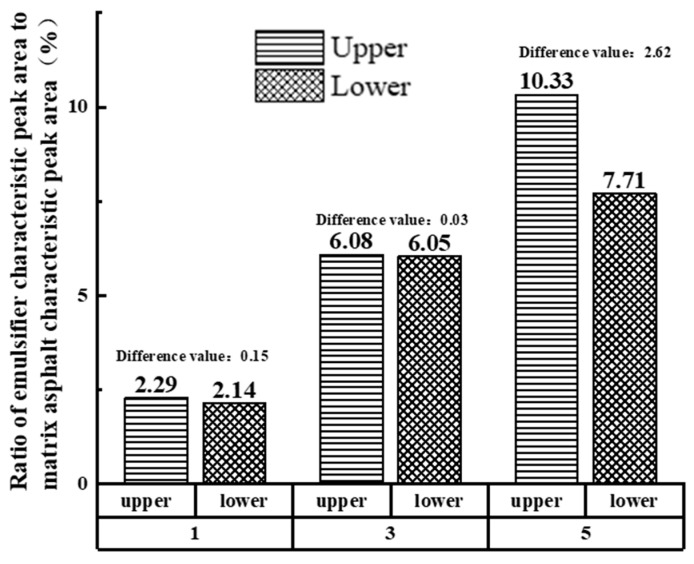
Proportion of emulsifier characteristic peak area in upper and lower branch tubes of storage-stabilized tubes.

To further compare the effect of different emulsifier admixtures on the storage stability of biomass asphalt/water emulsions, the difference in the ratio of the absorption peak area corresponding to the emulsifier of the evaporated residue of the upper and lower emulsions of the storage stability test tube to the absorption peak area corresponding to the matrix asphalt was used for evaluation. The absorption peaks corresponding to the matrix asphalt were selected as wave numbers 2948 cm^−1^, 2849 cm^−1^, 1455 cm^−1^, 1375 cm^−1^, etc., while the emulsifiers were selected as the most significant wave peaks 1151 cm^−1^, 1124 cm^−1^, 628 cm^−1^, etc. The characteristic peak area values and the different results are shown in Table 6 and Figure 11 [39,40].

As shown in Figure 11, the ratio of the characteristic peak area of the emulsifier to the characteristic peak area of the matrix asphalt in the upper and lower branches of the stability test tube tended to decrease and then increase with the increase of the emulsifier, which is similar to the results obtained from the storage stability test.

## 4. Conclusions

1. With the increase of emulsifier dosing, the peak of the radial distribution function of biomass asphalt/water emulsions tends to decrease and then increase, which is consistent with the storage stability experimental results; however, when the emulsifier content is too large, it will lead to the phenomenon of emulsifier agglomeration.

2. Emulsifiers can promote the mutual solubility of the asphalt–water phase, increasing the thickness of the interfacial layer between the two components, and the interfacial layer can prevent the aggregation of particles, thus ensuring smaller emulsion particles and enhancing the stability of the emulsion, but when the content of emulsifier is too high, the emulsifier forms micelles, resulting in an increase in the average particle size of the emulsion, and the emulsifier does not give full play to the role of enhancing the interaction of the asphalt–water phase, making the thickness of the interfacial layer between the two components smaller, and thus reducing stability.

3. Infrared spectroscopy analysis found that the infrared characteristic peak area ratio of emulsifier to matrix asphalt of the emulsion evaporation residue of the upper and lower layers of biomass asphalt/water emulsions with different emulsifier contents showed a trend of firstly increasing and then decreasing with the increase of emulsifier content, which is consistent with the stability performance trend derived earlier, indicating that the infrared absorption peak area ratio can evaluate the stability performance of biomass asphalt/water emulsions.

4. When biomass asphalt/water emulsion has the best emulsifier dosage, the emulsion can achieve the best stability performance, and finding the best emulsifier dosage of the corresponding emulsion can have high value for engineering applications, but the impact of emulsifier content on other properties of biomass asphalt/water emulsion is not explored in this paper; follow-up research should focus on the analysis of emulsifier content on the permeability of biomass asphalt/water emulsions and other road performance measures.

## Figures and Tables

**Table 1:** Conventional indexes of base asphalt.

	25 °C Penetration (0.1 mm)	25 °C Ductility (cm)	Softening Point (°C)	60 °C Brinell Viscosity (Pa·s)
**Test results**	63.7	>100	47	203
**Index requirements**	60–80	>100	>46	>160
**Test method**	GB/T0606-2011	GB/T0605-2011	GB/T0606-2011	GB/T0625-2011

**Table 2:** Proportion of four components of SK-70 base asphalt (%) [22].

Types of Asphalt	Asphaltene	Saturate	Aromatics	Resin
**SK-70#**	12.44	26.62	44.62	16.32

**Table 3:** Model parameters of 12-component base asphalt.

Components		Molecular Formula	Number of Molecules	Model Component(%)	Actual Component (%)	Proportion Error (%)
**Asphaltene**	Phenol	C_42_H_54_O	1	13.28	12.44	0.84
	Pyrrole	C_66_H_81_N	1			
	Thiophene	C_51_H_62_S	1			
**Aromatics**	Quinolinohopa-ne	C_40_H_59_N	2	26.93	26.62	0.31
	Thioisorenierat-ane	C_40_H_60_S	1			
	Benzobisbenz-othiophene	C_18_H_10_S_2_	3			
	Pyridinohopan-e	C_36_H_57_N	2			
	Trimethylbenz-eneoxane	C_29_H_50_O	2			
**Resin**	PHPN	C_35_H_44_	14	44.17	44.62	0.45
	DOCHN	C_30_H_46_	2			
**Saturate**	Squalane	C_30_H_62_	4	15.91	16.32	0.41
	Hopane	C_35_H_62_	2			

**Table 4:** Molecular parameters of other components of bio-oil asphalt/water emulsion.

Components		Molecular Formula	Molecular Weight (g/mol)	Number of Molecules	Ratio to Asphalt (%)
**Emulsifier**	1%	C_24_H_32_O_7_Na_2_S_2_	542	1	2.7
	3%			3	6.5
	5%			5	10.3
**H_2_O**	1%	H_2_O	18	904	97.3
	3%			867	94.1
	5%			826	89.6
**Biomass of oil**	Hexadecenoic acid	C_16_H_32_O_2_	256.4	1	9.1
	Linolenic acid	C_18_H_32_O_2_	280.5	2	
	Oleic acid	C_18_H_34_O_2_	282.5	2	
	Stearic acid	C_18_H_36_O_2_	284.5	1	

**Table 5:** Particle size of bio-oil asphalt/water emulsion with different emulsifier contents.

Material	Bio-Oil Asphalt/Water Emulsion		
Emulsifier Content	1%	3%	5%
**D_10_**	1.712	1.579	1.647
**D_20_**	2.013	1.793	1.899
**D_30_**	2.310	1.941	2.193
**D_40_**	2.439	2.197	2.407
**D_50_**	2.648	2.315	2.591
**D_60_**	2.901	2.487	2.797
**D_70_**	3.201	2.664	3.134
**D_80_**	3.798	3.012	3.287
**D_90_**	4.312	3.510	3.978
**Volumetric mean particle size**	2.917	2.416	2.736
**Span of particle size**	0.982	0.834	0.899

**Table 6:** Infrared characteristic peak area of evaporation residue of bio-oil asphalt/water emulsion with different emulsifier contents.

Wave Number (cm^−1^)	2948	2849	1455	1375	1151	1124	628
Control Group							
**Upper 1%**	693.693	431.286	779.685	137.132	26.114	16.332	6.988
**Lower 1%**	713.879	459.798	779.638	154.987	25.369	13.874	5.746
**Upper 3%**	681.125	402.146	720.987	120.126	30.999	32.007	53.365
**Lower 3%**	696.348	396.984	738.969	118.741	32.941	32.104	55.001
**Upper 5%**	642.153	431.123	790.258	132.110	86.212	64.988	51.214
**Lower 5%**	649.336	440.369	805.112	140.136	48.764	34.589	73.951

## Data Availability

All data, models, and code generated or used during the study appear in the submitted article.
